# Supplementary material for: Characterization of Antioxidant and α-Glucosidase Inhibitory Compounds of Cratoxylum formosum ssp. pruniflorum and Optimization of Extraction Condition
Source: Antioxidants (Basel). 2023 Feb 17;12(2):511. doi: 10.3390/antiox12020511 (PMC9952466; doi:10.3390/antiox12020511)
Supplement: Supplementary file 1 [file antioxidants-12-00511-s001.zip › antioxidants-2213921-supplementary.pdf]

Characterization of antioxidant and α-glucosidase inhibitory compounds of *Cratoxylum formosum* ssp. *pruniflorum* and optimization of extraction condition

Hee Won Ahn [1], Le Nguyen Thanh [2], Le Quoc Khanh [3], Se Hwan Ryu [1], Solip Lee [1], Sang Won Yeon [1], Hak Hyun Lee [1], Ayman Turk [1], Ki Yong Lee [4], Bang Yeon Hwang [1] and Mi Kyeong Lee [1,*]

[1]College of Pharmacy, Chungbuk National University, Cheongju 28160, Republic of Korea

[2]Department of Medicinal Chemistry Technology, Institute of Marine Biochemistry, Vietnam Academy of Science & Technology, Hanoi, 10000, Vietnam

[3]Hatinh Pharmaceutical Company (HADIPHAR), Hatinh 45000, Vietnam

[4]College of Pharmacy, Korea University, Sejong 47236, Republic of Korea

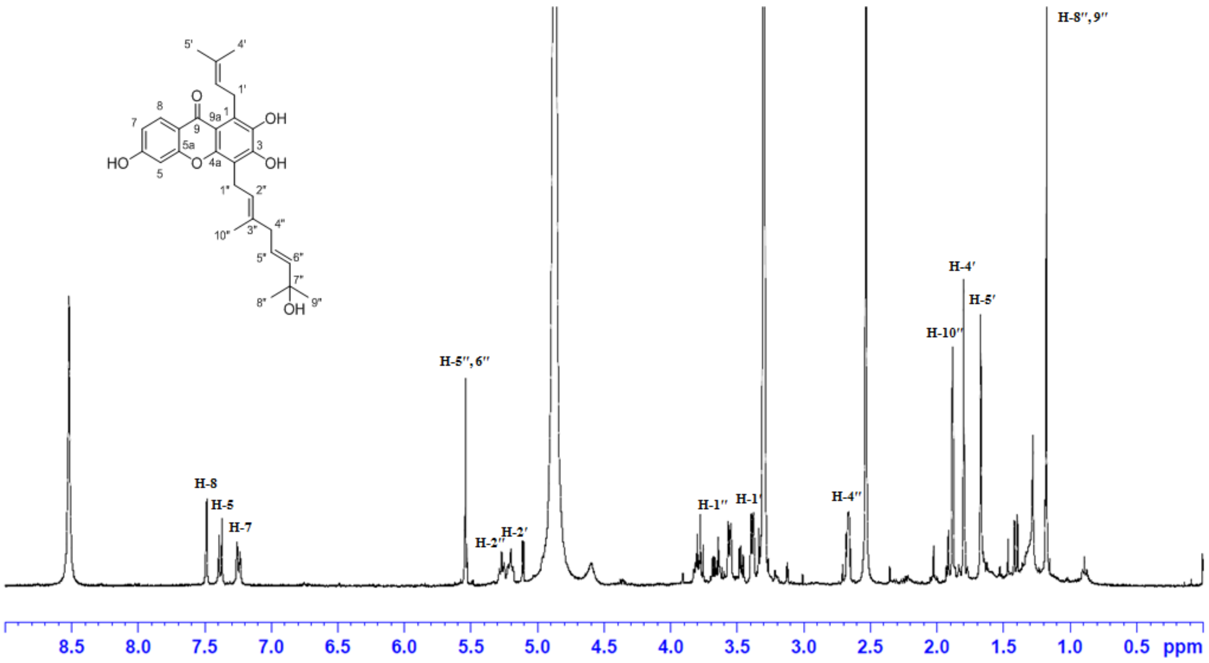

Figure S1. ^{1}H NMR spectrum of compound **16** (methanol-d_4, 400 MHz)

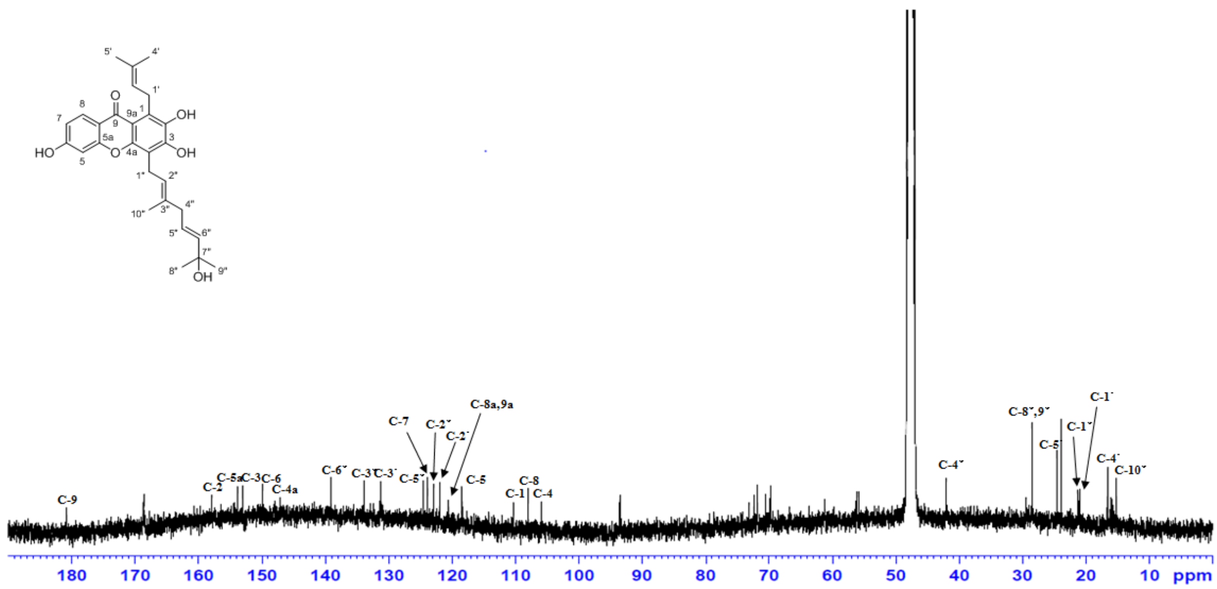

Figure S2. ^{13}C NMR spectrum of compound **16** (methanol-d_4, 100 MHz)

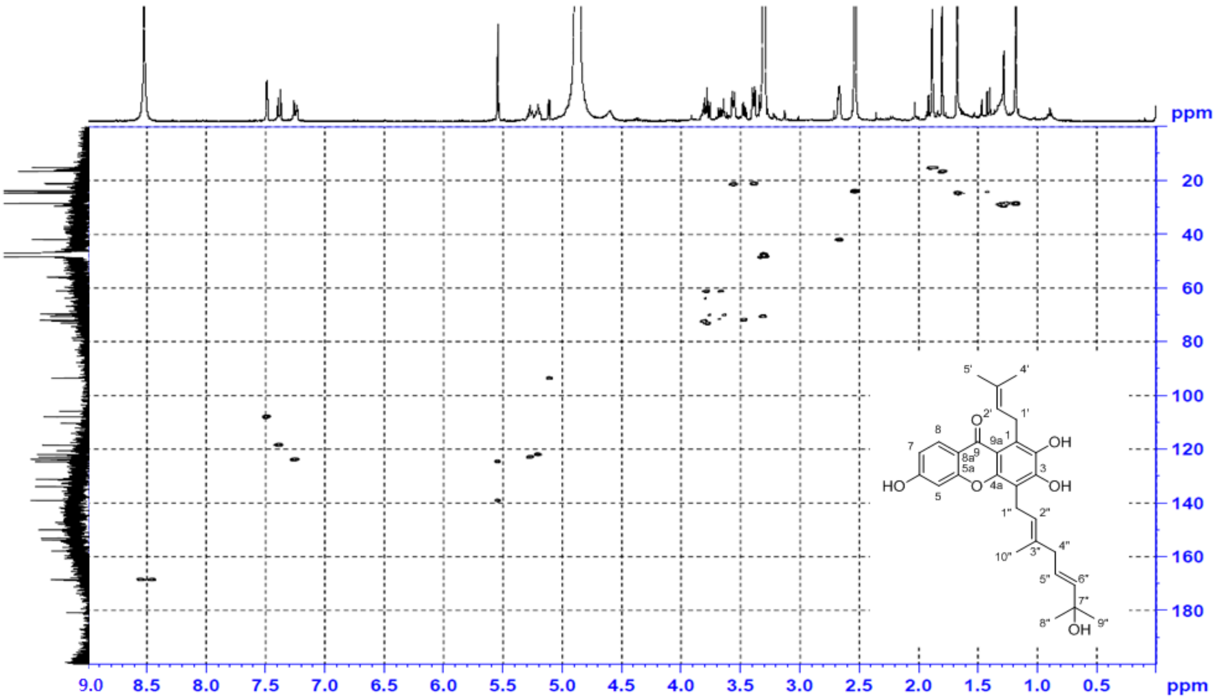

Figure S3. HSQC spectrum of compound **16.**

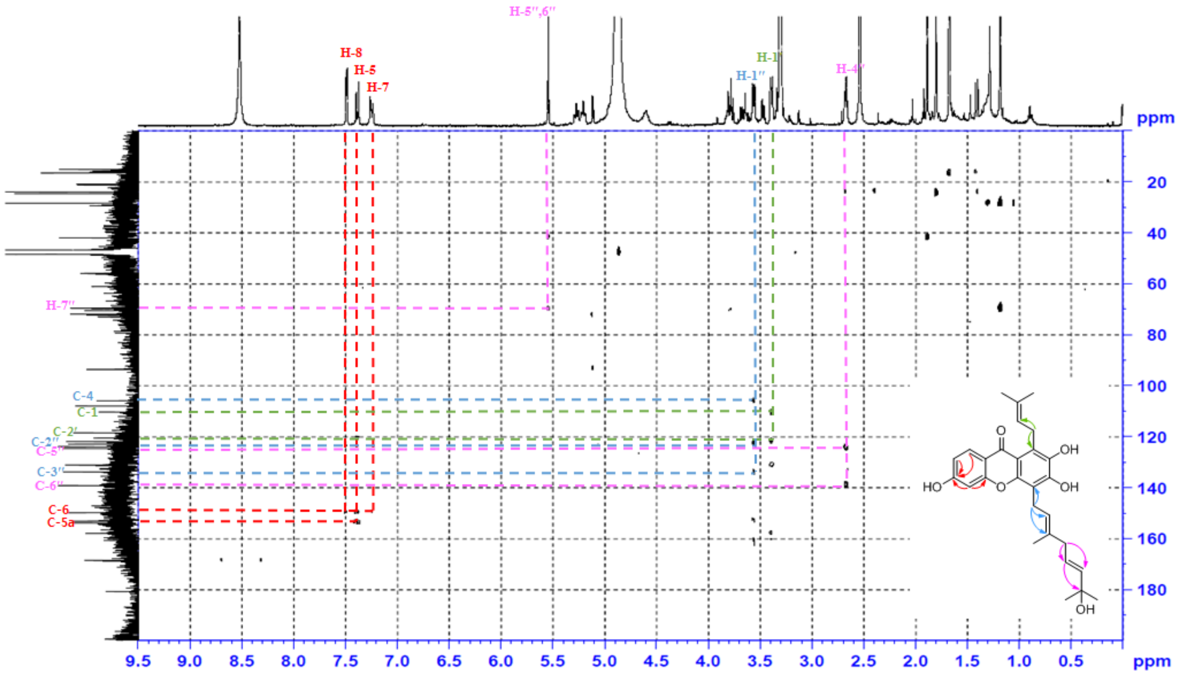

Figure S4. HMBC spectrum of compound **16.**

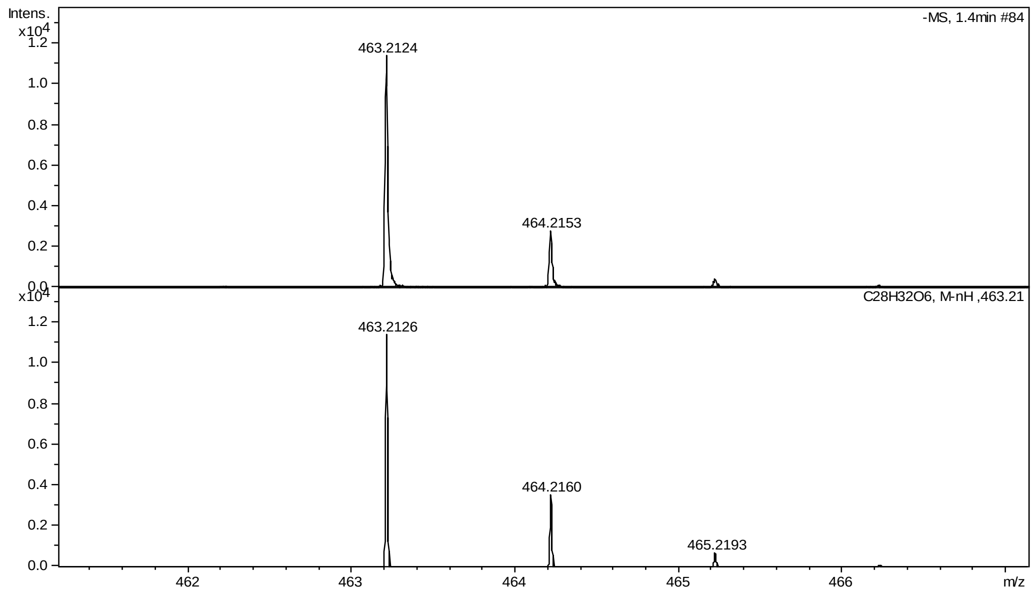

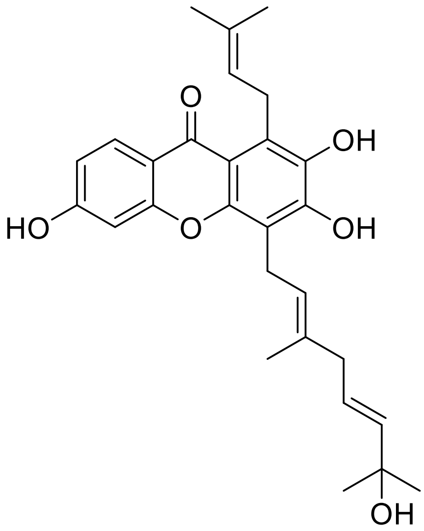

Chemical Formula: $C_{28}H_{32}O_6$

Exact Mass: 464.2199

Molecular Weight: 464.5580

463.2124 (Calcd. for $C_{28}H_{31}O_6^-$ 463.2126)

Figure S5. ESI-TOF-MS spectrum of compound **16.**

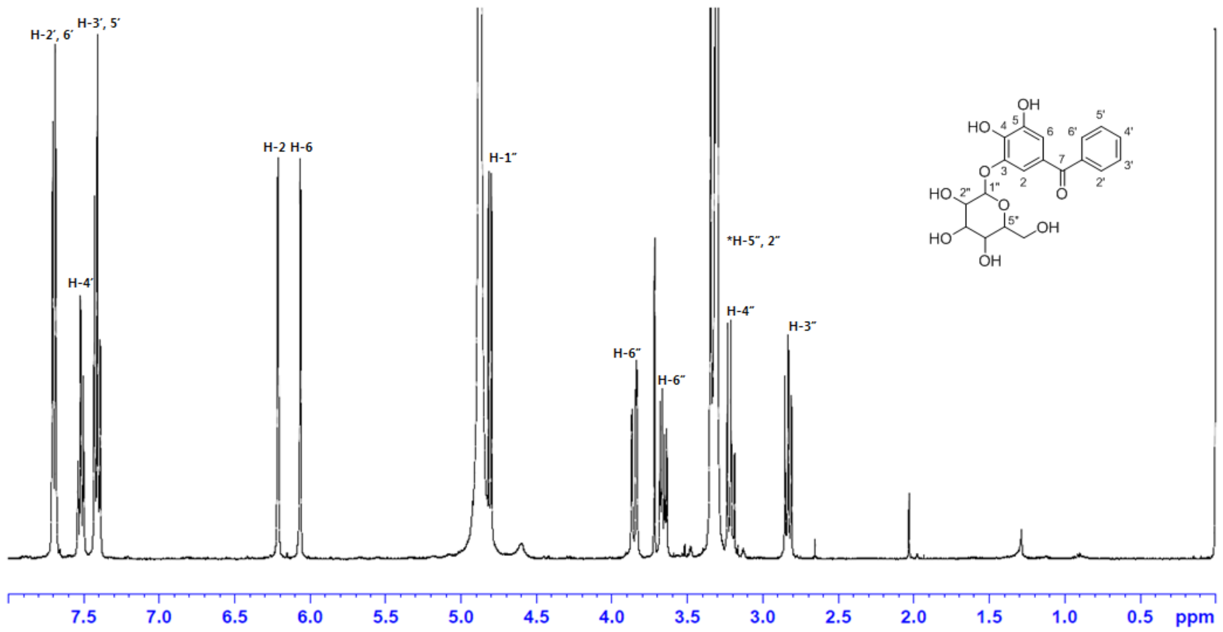

Figure S6. ^{1}H NMR spectrum of compound **19** (methanol-d_4, 400 MHz)

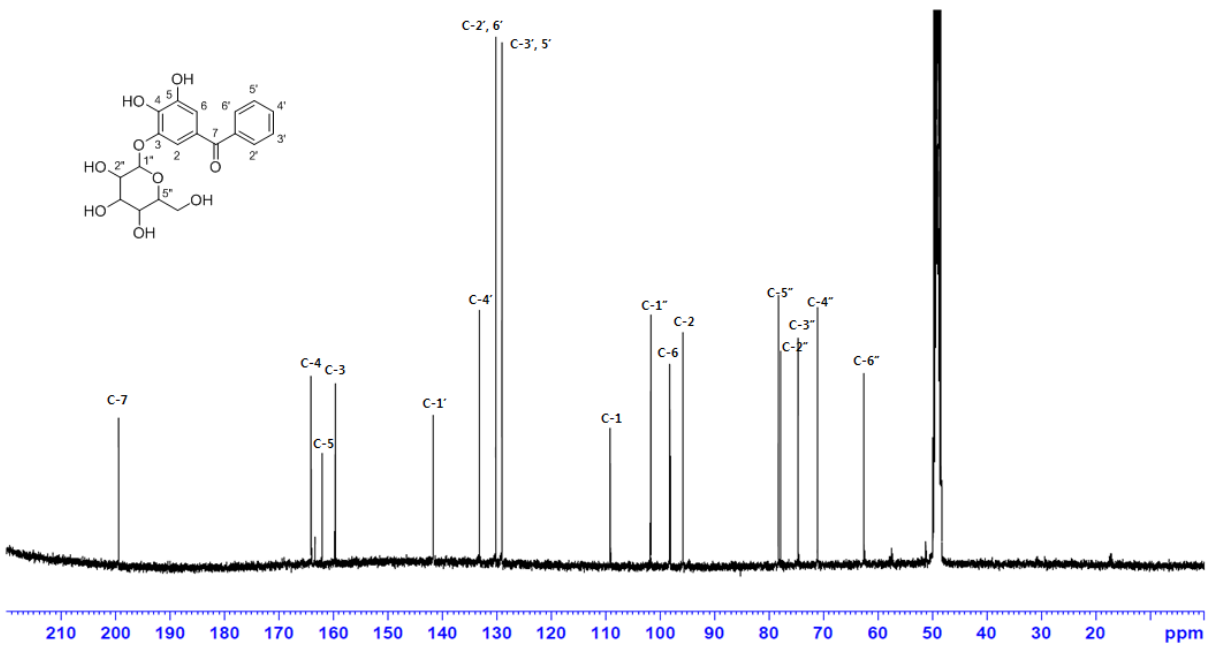

Figure S7. ^{13}C NMR spectrum of compound **19** (methanol-d_4, 100 MHz)

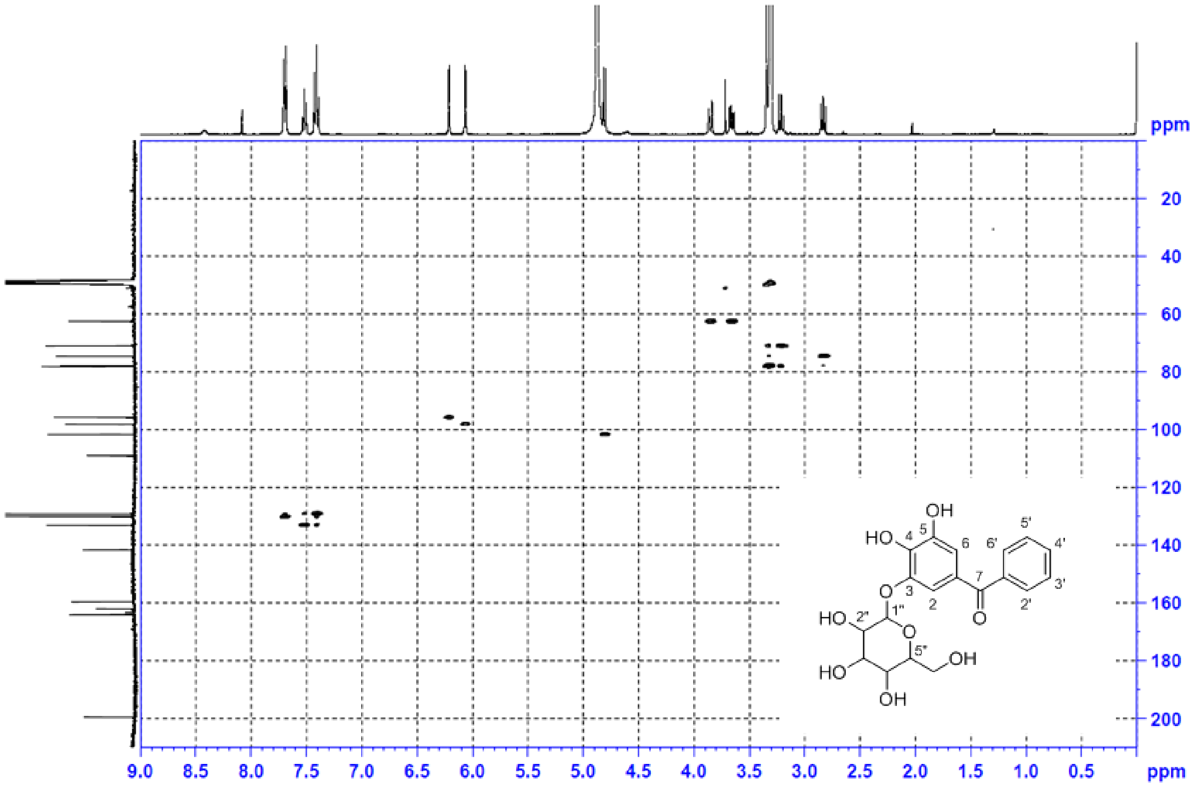

Figure S8. HSQC spectrum of compound **19.**

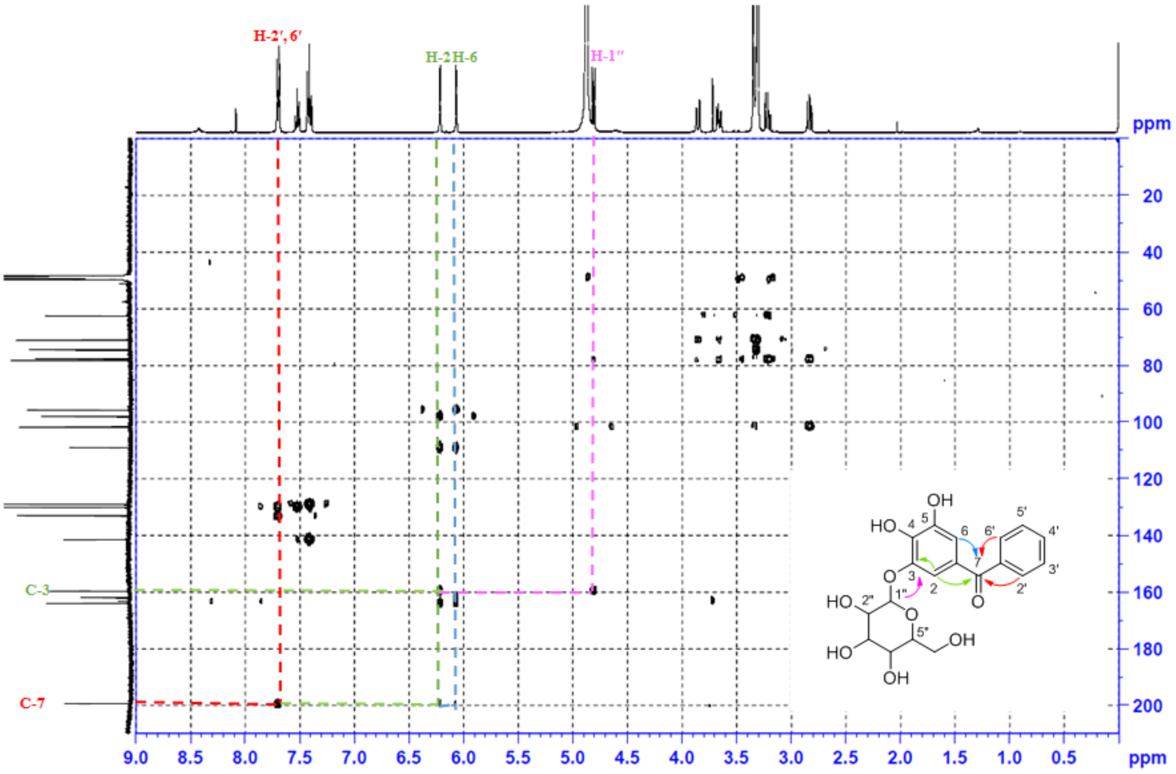

Figure S9. HMBC spectrum of compound **19.**

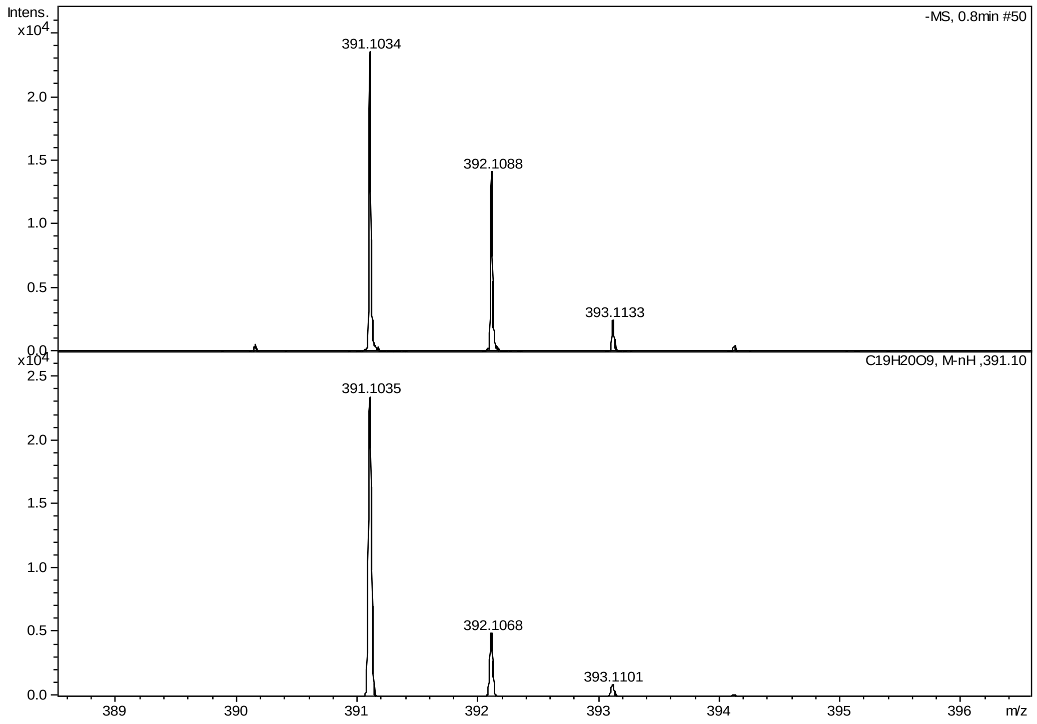

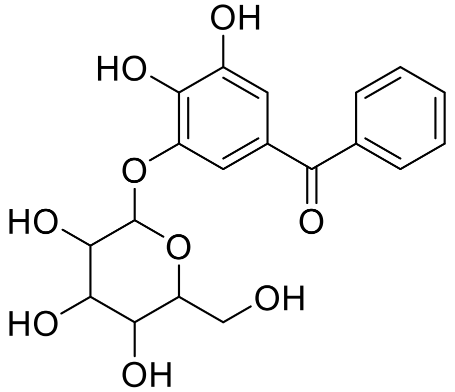

Chemical Formula: $C_{19}H_{20}O_9$

Exact Mass: 392.1107

Molecular Weight: 392.3600

391.1034(calcd. for $C_{19}H_{19}O_9^-$ 391.1035)

Figure S10. ESI-TOF-MS spectrum of compound **19.**
